# A Librarian's Visit to America

**Published:** 1965-04

**Authors:** A. E. S. Roberts

**Affiliations:** Medical Librarian, University of Bristol


### A LIBRARIAN'S VISIT TO AMERICA

BY

A. E. S. ROBERTS

*Medical Librarian, University of Bristol*

Thanks to the generosity of the Bristol Medico-Chirurgical Society and the University I was enabled to spend three weeks in America to attend the Second International Congress on Medical Librarianship in Washington and to see some American medical libraries.

I was one of a small party of eight librarians who sailed from Southampton on the *Queen Mary* on 6 June, 1963, and docked in New York on 11 June, where we were met by two American librarians who kindly conducted us to our hotel.

During the few days we were in New York we visited the medical library of Columbia University College of Physicians and Surgeons and the library of the New York Academy of Medicine.

The medical library of Columbia University is a vast library of some 250,000 volumes and nearly 4000 journals. There are also Neurology and Plastic Surgery branches, independent subject libraries in Psychiatry and Ophthalmology and many departmental libraries. Altogether these form the Medical Center Library, which represents the alliance of the Presbyterian Hospital and Columbia University. Since its founding in 1928, the Medical Center has had the three-fold objective of patient care, teaching and research. Because of lack of space in the College of Physicians and Surgeons building, books in the clinical sciences before 1930 and in the basic sciences before 1920, as well as journals before varying dates, are housed in two annexes. It takes 48 hours to get an item from these annexes for a reader. Photostats, microfilm, Xerox and translations may be obtained at reasonable prices. The hours of opening during term are 8.30 a.m. till 11 p.m. Mondays to Fridays, 9 a.m. till 5 p.m. Saturdays, and 2 p.m. till 10 p.m. on Sundays. Students, officers, staff and employees of Columbia University and the Columbia-Presbyterian Medical Center are privileged to borrow books and journals, and the period of loan is two weeks. Individual instruction on the use of the library is given upon request.

The New York Academy of Medicine, an independent association of physicians, makes available the resources of its library to the general public, as well as to its own Fellows and holders of library cards. This large library contains nearly 400,000 volumes and about 3000 journals, and is open from 9 a.m. till 9 p.m. Mondays to Fridays, and 9 a.m. to 5 p.m. on Saturdays, but the general public may not use the library after 5 p.m., the evenings being reserved for Fellows only. Readers coming to the library are given aid in finding their material. The card catalogue and general and special indexes are explained whenever requested. Brief information is furnished by telephone, but it is urged that long, and particularly statistical queries be made by post or in person, since errors are thus likely to be less. The verification of references by telephone is likewise not recommended: it is safer to have these in writing also. The general aim is to assist the inquirer to locate what he wants to read, not to read it for him. Books may be borrowed for two weeks by Fellows and library subscribers. Unbound material cannot be borrowed.

I met other British librarians who had arrived in America before me, and we took the opportunity of seeing some of the sights of New York before leaving for Washington for the Congress, which was the main purpose of our visit.

We stayed at the Shoreham Hotel in Washington; and about one thousand medical librarians from sixty-two countries, including nearly forty from the United Kingdom, attended the Congress. The announced purpose of the Congress was "to foster the development and improvement of medical library service throughout the world", and sessions were held under such headings as "Education and training for medical librarianship", "Utilization of machines for bibliographical purposes", "Problems of library organization", and "Library co-operation".

Under the first heading it was said that the most desperate need in the general shortage of librarians was for librarians in small libraries. Many small libraries are managed by unqualified personnel, often anxious to learn and improve their performance. In parenthesis, I may say that the same situation obtains here in the south west of England, and to help alleviate the position I have agreed to hold a week-end workshop session in the near future for those keen people who have been placed in charge of small hospital libraries in the region. This idea of establishing local programmes of training in the basic elements of library practice was put forward at the Congress. Such courses have successfully been held by the American Hospitals Association, as well as by other authorities, and the response has been overwhelming. In British Columbia a scheme is in operation whereby a travelling librarian gives basic instruction to those who have charge of small libraries in remote areas.

A number of papers were given on the utilization of computers for bibliographical purposes. A computer has been developed and installed at the magnificent, newly built National Library of Medicine at Bethesda, near Washington, both for the storing and retrieving of information and for printing. The scheme (MEDLARS it is called—Medical Literature Analysis and Retrieval System) can both give out bibliographical citations on a given subject (but only from 1963) and print the *Index Medicus*. This mechanization of the *Index Medicus*, which has been going on since the beginning of 1964, means that the speed of production of the *Index Medicus* and the coverage of medical literature will be increased. In the not-too-distant future, MEDLARS will be dealing with 250,000 citations annually; at present the number is about 150,000. In 1965, in addition to the journal articles, the *Index Medicus* will commence including monographs and later on the contents of symposia and congresses.

In the session on the problems of library organization, there was a paper on the setting up of a new medical school library in which consideration of the proximity of general library facilities and good relations with the local medical community were stressed. It was also stated that within the past eight years four new medical schools had been established in the United States and that there were plans in the offing for at least ten more in the United States and Canada. In a paper on helping the small libraries it was suggested that the small hospital library should emphasize service rather than resource-building; that is to say, it should frankly recognize dependence upon other libraries.

With regard to interlibrary co-operation we learnt that interlibrary lending in the Scandinavian countries is considered one of the most positive contributions any research library can make to the national research programme. The free flow of material from one library to another is facilitated by the franking privilege accorded to state-financed institutions in Sweden. Microfilm and photocopying are largely used in interlibrary loans. The enormously rising production of scientific literature demands a co-operation which extends also to the policy of acquisition, at any rate for the less-used medical periodicals. In the United States the same problems arise with interlibrary loans as were met with ten years ago, i.e., lack of uniformity in requests, in period of loan, in type of material loanable, and also in completeness or accuracy of references. The National Library of Medicine, however, has so improved its interlibrary scheme that it is not surprising that this department is very heavily burdened. Because of Federal support, it is able to supply free photocopies to libraries. It ceased to lend to individuals in 1957, because of the abuse of the scheme of supplying free photocopies of articles, and lends only to libraries.

After the Congress I went to Boston by air to see the Boston Medical Library and the Harvard Medical Library. These two libraries are about to amalgamate to form the Francis A. Countway Library at Harvard in a magnificent new library building which was just beginning to be built when I was there. In the drawing up of the plans for this eight-storey building, the librarians exercised the main influence. On the ground floor will be the less-used journals, on the first floor the last ten years of the most used journals, on the second floor, which will be the main entrance, will be reference, catalogues and staff accommodation, on the third floor will be monographs published during the last ten years, on the fourth and fifth floors will be earlier monographs, the sixth floor will be devoted to the history of medicine and club rooms, and the top floor will house the editorial offices of the New England Journal of Medicine and the Journal of Bone and Joint Surgery. In the existing Harvard Medical Library there is a coin-operated photostat machine. The library is open till 10 p.m. Mondays to Fridays, till 5 p.m. on Saturdays, and from 2 p.m. till 6 p.m. on Sundays.

While in Boston we also visited the Treadwell Library in the Massachusetts General Hospital. There was a time, not too long ago, when it was possible for quite a modest medical library, even in a large hospital, to meet with reasonable success the needs of the doctors on the hospital staff. It is a different story nowadays, but medical libraries in hospitals have not developed to meet these very different needs. But the excellent Treadwell Library is an example of a hospital library that has attempted to grow with the times and has received fiscal support in this attempt. It contains 25,000 volumes and subscribes to 500 journals.

From Boston we went by train to Montreal to see the McGill Medical Library and, of course, the Osler Library. The McGill Medical Library, founded in 1823, is a library of some 100,000 volumes and 1300 journals, and is the only one among Canada's thirteen medical school libraries that reaches the U.S. Public Health Service minimum size for medical school research collections. The Osler Library, hallowed place, is a special library of the history of medicine and science, and comprises some 13,000 volumes. It occupies a large room, beautifully equipped, convenient to the medical library.

We spent three very pleasant days in Montreal and then flew back to London.

The American medical libraries I saw are on the whole much larger than ours in content, staff and budget, but a great number are badly housed in that they all seem to be bursting at the seams. The interlibrary loan department at Harvard is accommodated on a staircase! Most of the medical school libraries suffer from an acute lack of work space. It is said that American medical school libraries double in size every 12 years. Library service is paramount with American medical librarians; in the large libraries the staff is highly qualified and numerous enough to offer bibliographical, abstracting and even translation services.

Considering the large number of periodicals taken by some of the libraries, sometimes 2000 titles or more, it seems to be a reflection on the supposed need for such a large number of journals when it is stated that 262 of these titles in one library accounts for 82 per cent of use of journals, and of these 67 accounted for 50 per cent of the loans of recently published issues. That we possess nearly all these 67 amongst our mere 500 titles shows how selective are the journals we choose to take in our own library. The larger the library the more complicated it is both to use by the reader and to administer by the staff, and the staffs in the large American libraries are very numerous, necessarily so. An item should not be added to a library just because it has been published; whether or not it is likely to be used should be the criterion of selection. The huge distances between some libraries in the United States and Canada is a good reason, of course, for unlimited acquisition.

There were many delightful informal occasions during my visit, and none more so than the picnic at Stone Lake, near Washington, given by the Naval Medical Center, when several hundred of us were served with hot dogs, fried chicken, potato salad, etc. and iced beer. But I think the deepest impression made on all of us was the genuine friendliness and universal hospitality of our American hosts, many of whom welcomed delegates to their homes. I myself enjoyed many such invitations, including a week-end with one host. The immense amount of preliminary work of arranging the Congress, and the ever-present concern of the organizers for our comfort are things we will never forget. And the highlight for us all was undoubtedly the visit to the White House, when President Kennedy kindly addressed us. It was truly a memorable occasion.

